# Whole Transcriptome Sequencing Unveils the Genomic Determinants of Putative Somaclonal Variation in Mint (*Mentha* L.)

**DOI:** 10.3390/ijms23105291

**Published:** 2022-05-10

**Authors:** Felipe López-Hernández, Andrés J. Cortés

**Affiliations:** Corporación Colombiana de Investigación Agropecuaria (AGROSAVIA)-CI La Selva, Rionegro 054048, Colombia

**Keywords:** *M. × piperita* L., *M. spicata*, RNA-Seq, comparative transcriptomics, crop biodiversity

## Abstract

Mint (*Mentha* L., Lamiaceae) is a strongly scented herb of the family Lamiaceae that is grown mostly by clonal propagation, making it a valuable species for the study of somaclonal variation and its phenotypic consequences. The recent introduction of a few species of mint in South America, followed by a presumably rampant propagation, make this region particularly ideal for studying the extent of somaclonal genetic diversity. Hence, the objective of this work was to offer a preliminary characterization of somaclonal genetically coding diversity of the mint in the northern Andes in order to address the question of whether somaclonal variants may have emerged despite relatively recent introductions in a region where mint is not native. A total of 29 clonally propagated specimens, collected in mint export farms in the province of Antioquia, a major region for mint production in the northwest Andes of Colombia, were genotyped using RNA sequencing (RNA-Seq). SNP calling was carried out from the leaves’ transcriptome profiles of each plant by combining the GATK4 and TRINITY protocols, obtaining a total of 2033 loci across 912 transcripts with a minimum read depth of 20X and 4% of missing data. Unsupervised machine learning algorithms considered the *K*-means, AGNES and UPGMA approaches, all of which suggested three genetic clusters for *M. spicata* and a unique cluster for *M. × piperita*. The results indicate that at least two different origins of *M. spicata* reached the eastern region of the Antioquia province, clonally propagated in the locality ever since for local consumption and export. One of these ancestries had more population structure, possibly due to environmental or anthropological pressures that intervened in the fragmentation of this genetic group or to a higher somaclonal mutation rate. This work offers a first step into the study of the accumulation and transmission of presumably quasi-neutral somatic mutations at coding regions in an herbaceous clonally propagated scented species such as mint, likely favored by an expected population expansion after its Andean introduction. These ad hoc hypotheses warrant further study as part of future research.

## 1. Introduction

The rate, extent and architecture of de novo mutations and somaclonal variations have been long-standing intriguing questions in molecular evolution [1]. Genomic features and chromosomal constraints are well-known determinants of hidden levels of genetic variation [2]. Unusual segregation rearrangements and high ploidy levels are also regarded as enhancers of the nucleotide diversity at evolutionary timescales [3]. Yet, somaclonal variations at shallower timeframes have remained elusive [4], partly due to their presumed rarity and difficulty targeting them in non-model species. Besides, they are often assumed neutral, a corollary of the neutral theory of molecular evolution [5], so their potential adaptive or co-opted, s.s. [6,7], values have seldom been tested.

Crops maintained by clonal propagation constitute unique experimental playgrounds to target somaclonal variants and explore their phenotypic consequences. In particular, crops grown for their non-reproductive organs have severe disruptions to their flowering and fruiting systems, making clonality a compulsory propagation strategy. Mint (*Mentha* L.), a strongly scented herb of the Lamiaceae family, is an example of this. Several phylogenetically intricate species [8] have been used for centuries for medicinal and savory purposes, including 30 species and hybrids that are distributed or introduced throughout the globe [9]. Beyond their use as herbs and spices and for pharmaceutical needs, distilling of essential oils (i.e., menthol) from commercial peppermint is now a major global economic commodity. Yet, its bio-economical potential remains enormous due to other secondary metabolites and novel uses from biomass waste.

Despite mint’s cosmopolitan native range, the only subcontinent where they had to be introduced as part of the Colombian interchange was South America. Recent introductions of a few mint species [9], followed by their clonal propagation, make this region ideal for studying the extent of somaclonal genetic diversity. However, complex genomes, polyploidization and hybridization within the genus [8] make mandatory the use of transcriptomics to target somaclonal variation in allelic coding variants at higher resolution. Comparative works [10] suggest that the most efficient pipeline to reconstruct the matrix of allelic variants from RNA sequencing is the integration of the TRINITY algorithm as a de novo assembler [11] and the GATK4 protocol for SNP calling [12,13]. Hence, running the integrated TRINITY + GATK4 protocol has allowed recovering SNPs with 100% accuracy in cases such as with peaches and tangerines [10]. Yet, this protocol has not yet been tested for highly scented clonal herbs such as mint as a strategy to explore somaclonal variants in expressed regions. Given this research gap, this study’s goal was to carry out a preliminary assessment of coding somaclonal diversity in mints from the northwest Andes using RNA sequencing (RNA-Seq) and the combined TRINITY + GATK4 protocols. Specifically, we wondered (1) whether somaclonal variation may have arisen despite relatively recent mint introductions to a region where the species is not native and (2) whether these variants may have any phenotypic effect. Exploring these questions offers a first step to unveiling the role of molecular evolution and molecular genetic resources in the plant improvement of clonally propagated crop species, just as envisioned by the International Journal of Molecular Science’s special issue on Molecular Genetics and Plant Breeding. Diversity trends defined here will serve as null hypotheses to test underlying mechanistic molecular and biochemical processes.

## 2. Results

Variation across 29 specimens of mint sourced a matrix of allelic variants using the supertranscript and the hybrid protocol GATK4 + TRINITY for RNA-Seq. We used this SNP information to carry out a principal component analysis (PCA) as input for partitional and hierarchical clustering approaches. Additionally, we carried out a distance-based analysis using a UPGMA dendrogram. All these algorithms suggested a clear difference between *M. spicata* and *M. × piperita*, which validates the use of *M. × piperita* as a control group for the exploration of the somaclonal genetic diversity within *M. spicata*. The optimization in the number of clusters and the UPGMA dendrogram suggested the presence of three possible groups of *M. spicata* and one of *M. × piperita*.

### 2.1. The Commercial Protocol Recovered Greater Quantity and Quality of RNA

Among six RNA extraction protocols, the Qiagen^®^ RNeasy Plant Mini Kit commercial kit allowed us to obtain the best concentration quality and A260/A280 and A260/230 absorbance ratios. In this sense, this commercial protocol was scaled to the 29 specimens (Table S2), conveying optimal RNA concentration and quality as follows: mean Nanodrop^®^ and Qubit^®^ concentrations of 922.021 ug/uL (IC: 236.52) and 94.79 ug/uL (CI: 8.78), and mean A260/280 and A260/230 ratios of 2.11 (CI: 0.016) and 2.02 (CI: 0.23).

### 2.2. Sequencing and Data Cleaning

The 29 genetic libraries built for RNA-Seq had a mean Qubit^®^ concentration of 18.77 ug/uL (CI: 5.5431), a mean fragment size of 282.66 bp (CI: 3.099), and a quantification TapeStation^®^ mean of 99.96 nM (CI: 29.165). For all specimens, electropherograms suggested fragment distributions with defined peaks and the absence of contaminants (Table S3). After the trimming for each specimen by the Trimmomatic algorithm, all samples had quality scores greater than 30 using 1.9 Illumina encoding (Figure S1A) without the presence of adapters (Figure S1D), high duplications percentage (Figure S1B) and GC deviation percentage to be expected for RNA-Seq data (Figure S1C).

### 2.3. Supertranscript of Mint as Reference for SNP Calling

A supertranscript was assembled as guiding reference for alignment of reads and SNP calling across all 29 specimens of mint (Table 1).

De novo transcriptome assembly used TRINITY software from all trimmed *fastq*. It was composed of 509,754 transcripts (Figure S2A) with an average length of 557.9 bp, a minimum length of 178 bp, a maximum length of 12,186 bp and a GC percentage of 43.2%. The transcriptome presented splice isoforms that could increase the false-positive rate in calling allelic variants. Thus, using the transcriptome and trimmed *fastq*, we built a supertranscript collapsing regions of unique and common sequences between splice isoforms in a unique linear sequence (Davidson et al., 2017). This led to a supertranscript of 352,512 transcripts, with an average length of 472.2 pb, a minimum length of 201 bp, a maximum length of 15,765 bp and a GC percentage of 43.1% (Figure S2B).

### 2.4. A Total of 2033 SNP Markers Were Recovered in 912 Transcripts with Depth of 20X

We made a script to automate the process for SNP calling based on the GATK4 protocol (raw data, input files and bioinformatic scripts are available in https://github.com/FelipeLopez2019/RNAseq-SNP-Calling-GATK4-Mint). A mean of 15,588,705 reads (IC: 1,368,899) were obtained in all 29 specimens, of which 15,136,482 (IC: 1,403,825) were mapped to the supertranscript, with a mapping percentage of 96.81%. We obtained a high number of duplicated reads in the mapping, with a mean of 11,257,168 reads (IC: 1,166,846). In order to avoid confounding paralogous transcripts with allelic variants, duplicated reads were removed in the intermediate steps of the GATK4 protocol, obtaining a mean of 3,879,314 reads (IC: 293,552) without duplicates for all 29 specimens (Table 1). After that, we extracted a gVCF for each specimen using the function *HaplotypeCaller*, and we collapsed these gVCFs to a global VCF. This resulted in a matrix of allelic variants with 2033 loci in 912 transcripts for all specimens, with a minimum read depth of 20X and a maximum missing data percentage of 4%.

### 2.5. M. spicata Clones Were Distributed in Three Genetic Groups in the Northwest Andes

With the aim of reconstructing mint’s genetic variability in the northwest Andes, we carried out clustering analyses following two families of algorithms, hierarchical and partitional methods, both of which used the principal components from the PCA analysis. Moreover, we performed visualization by means of unsupervised dendrogram clustering reconstructed using the UPGMA analysis. All methods suggested the presence of three genetic groups of *M. spicata* and a unique group for *M. × piperita*.

**Figure 1:**
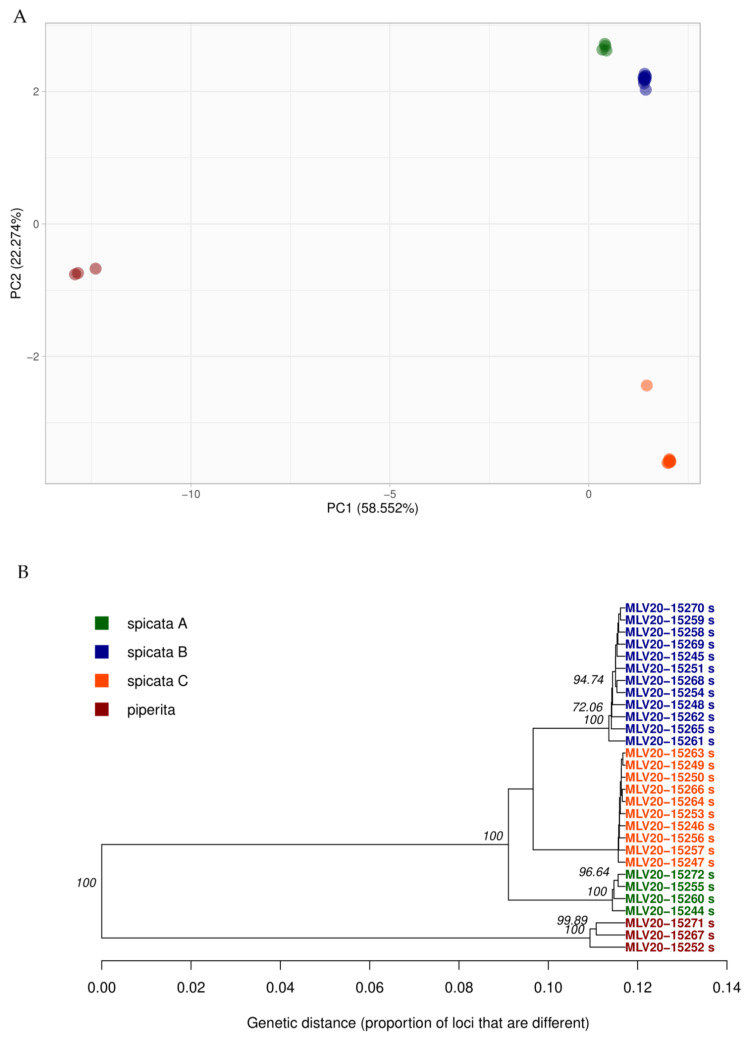
Overall diversity patterns in mint from the northwest Andes. (**A**) PCA analysis from 2033 loci distributed in 912 transcripts. The first component explained 58.56% of the variance, and the second component explained 22.27% of the variance (both totaling 80.83% of explained variance). Clustering validation was performed using the algorithms *NbClust* and *optCluster*, which suggested a total of four clusters: three of *M. spicata* (spicata A, spicata B and spicata C) and one of *M. × piperita*. All clusters were recovered by the first two components. (**B**) Dendrogram carried out by UPGMA analysis from 2033 variants distributed in 912 transcripts using Nei’s distance and bootstrap as resampling method with 10,000 replicates. These results also suggested a total of four clusters: three from *M. spicata* (spicata A, spicata B and spicata C) and one for *M. × piperita*. Clusters under both approaches in (**A**) and (**B**) are fully concordant.

Specifically, from the 2033 loci distributed in 912 transcripts, we carried out a dimensional reduction to principal components. The first component explained 58.56% of the overall variance, the second component explained 22.27% of the variance and the third component accounted for 11.28%. We further performed clustering validation with the *NbClust* y *optCluster* algorithms. Both suggested a total of four clusters: three from *M. spicata* (spicata A, spicata B and spicata C) and one to *M. × piperita* (Figure 1A). All four clusters were recovered with the first two components, which together accounted for 80.83% of the overall variation, overpassing the 80% threshold. On the other hand, *optCluster* suggested that the best hierarchical algorithm to reconstruct the clustering was AGNES, and the best partitional algorithm was *K*-means. Both approaches tagged specimens identically. In the same way, the UPGMA approach suggested three groups for *M. spicata* (spicata A, spicata B and spicata C) and one for *M. × piperita* (Figure 1B).

### 2.6. Gene Functionality of Polymorphic Transcripts

All 2033 variants distributed across the 912 transcripts spanned both *M. spicata* and *M. × piperita* (Figure S3). From all 912 transcripts, 96.10% retrieved hits in *BlastX* using the software *Blast2GO*. The 84.57% of these Blast *hits* were mapped, of which it was possible to annotate 94.27% with GO and enzyme codes (Table S4). Using the GO codes, we constructed three pathways to analyze the frequency of biological, cellular (Figure S4) and molecular (Figure S5) processes, as detailed below.

**Figure 2:**
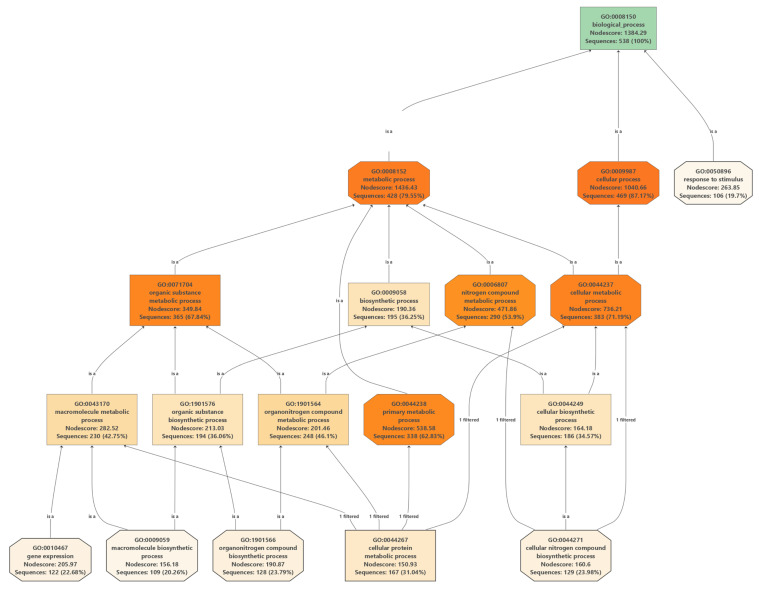
GO pathway to analyze polymorphic biological processes via GO codes from *Blast2GO*. GO terms ranged from 35,822 to 1471.06 and the number of associated transcripts ranging from 365 to 428 sequences. Main GO terms related to carbohydrate and energy metabolism (GO:0044237 and GO:0044238) and cysteine and methionine synthesis (GO:0008152 and GO:0009987). We also explored the enzyme codes of the 52 KEEG pathways associated with all polymorphic transcripts (Table 2, extended in Table S5). Of the KEEG pathways, 25% were related to carbohydrate metabolism, 19.23% of the KEEG pathways were related to amino acid metabolism, 11.54% of the KEEG pathways were related to energy metabolism and the other pathways were associated with less than 10% of the target queries. Main pathways of carbohydrate/amino acid synthesis were linked to glycolysis/gluconeogenesis (Figure S6), and cysteine/methionine metabolism (Figure S7) [14].

We first explored the top GO terms in each pathway using the high *Nodesores* and number of associated transcripts. In the biological pathway (Figure 2), we analyzed the GO terms in the *Nodescore* range from 35,822 to 1471.06 and the number of associated transcripts ranging from 365 to 428 sequences. The main GO terms were related to proteins of carbohydrate and energy metabolism (GO:0044237 and GO:0044238) and the synthesis of cysteine and methionine metabolism (GO:0008152 and GO:0009987) (Table S4). In the molecular pathway, we analyzed the GO terms in the *Nodescore* range of 626.86 to 704.47 and the number of associated transcripts ranging from 349 to 380 sequences. The main GO terms were related to proteins with catalytic activity, biosynthetic process and regulation of transcription (GO:0003824) (Table S4). On the other hand, for the cellular pathway, we analyzed the GO terms in the *Nodescore* range of 762 to 1224.97 and the number of associated transcripts ranging from 305 to 409 sequences. GO terms related to proteins linked in intracellular anatomical structure (GO:0005622) (Table S4).

## 3. Discussion

Recent introductions of a few mint species in South America as part of the Colombian interchange, followed by presumably rampant clonal propagation, make this region ideal for studying the extent of putative somaclonal genetic diversity. In addition, the use of transcriptomics’ profiles makes it possible to target variation in allelic coding variants at higher resolution while reducing the complexity of correctly aligning polymorphic non-coding regions and highly repetitive regions. Our results suggest that despite mint having relatively recent introductions to the northern Andes, where it is not native, coding variants were detected across all samples spanning a total of three groups for *M. spicata* and one group for *M. × piperita*, as well as putative somaclonal variation within clusters. The candidate somaclonal variants may be attributed to primary metabolic pathways, suggesting a likely predominant role of silent mutations, with sporadic co-opted variation as part of mint’s colonization of the northern Andes (e.g., to shorter day length and unforeseen pressures by local populations of herbivores).

### 3.1. Origins and Extent of Genetic Clusters and Putative Somaclonal Variation in Mint

Gene ontology and KEGG analyses suggest a predominance of silent mutations, likely hidden from purifying selection [15]. However, novel mutational variants in defense transcripts may also speak for some co-opted (s.s. [6,7]) adaptive variants as part of human-mediated mint’s colonization of the northern Andes, exposing introduced genotypes to shorter day lengths and unexpected antagonist biotic interactions. In order to discern the relative role of neutral and mutational load better, as part of a revisited neutral theory of molecular evolution [5] within a somaclonal framework, future studies should envision building a more compelling resource of somaclonal variance, as well as their timing of appearance. For instance, complete re-sequencing of the genome, with a careful phase reconstruction and purge of highly repetitive regions, could clarify both the fine-tuning of the three mint groups and the concrete metabolic pathway correlates beneath the candidate somaclonal variation.

### 3.2. Utility of RNA-Seq to Study Presumably Adaptive Variation

Capturing somaclonal variation from Whole Genome Re-sequencing (WGR) would be the most sensitive method. However, within the genus Mentha, there is only one genome annotated for horsemint (*M. longifolia*) with a genome size of 468,947 Mb, and *M. spicata* is an orphan species from a genomic perspective that does not yet have an annotated reference genome at the chromosome level. Producing this resource de novo would generate additional efforts, raising laboratory and computing costs. Meanwhile, genotyping based on reduced representation libraries, such as Genotyping-by-Sequencing (GBS), would not have the necessary resolution to capture somaclonal variability because it may be enriched in repetitive regions or conserved genes with little change to exhibit somaclonal segregation. This is why a more reliable alternative for a genus with complex genomes, repeated polyploidization and rampant hybridization [8] is transcriptomics, which nowadays is supported by modern RNA-Seq methodologies widely used to carry out expression analysis. In this sense, the present study was able to explore a de novo RNA-Seq strategy using the TRINITY assembler to obtain a supertranscriptome [13], which served as a reference in the calling of variants.

Still, there are few studies testing different methods for the optimization of allelic variant calling from RNA-Seq. Therefore, we complemented the pipeline with recent recommendations for fruit tree species such as mandarin, in which authors reported a viable integration between the TRINITY and the GATK4 algorithms ultimately to increase the number of allelic variants captured while efficiently controlling for false positives. As a control species within the integrated pipeline, a peppermint accession was sampled under the same environmental conditions, given that it is also largely used to export from the northwestern Colombian province of Antioquia, east high plateau.

We were further able to confirm the requirement to bring the genotypes to a homogeneous greenhouse climate before sampling leaves for RNA-Seq sequencing. Otherwise, somaclonal variation may have been unbiased across transcripts and samples, precluding a systematic understanding of the different metabolic pathways prone to putative somaclonal variants. Preliminary greenhouse treatments enabled gene ontology and KEGG analyses to suggest that coding variation is more likely to appear in transcripts associated with primary metabolism (glycolysis/gluconeogenesis), synthesis of essential biomolecules and defense compounds (cysteine and methionine pathway) [14].

### 3.3. Caveats and Perspectives

Given the pilot nature of the present research to assess coding variation in a clonally propagated species such as mint, explicitly recognizing the study’s limitations will guide future efforts to examine more rigorously some of the proposed ad hoc hypotheses. Among these, useful reference questions worth testing in more detail as null hypotheses in future research are: Did genotypes actually reach homogeneous acclimatization after the greenhouse treatment? What is the factual role of the presumed population expansion of mint linages after its Andean introduction? What is the cause of greater population structure in one of the *M. spicata* founding clades? Are putative somatic mutations genuinely quasi-neutral? What are their key phenotypic functional effects?

We must also acknowledge the fact that the assumption of the narrow founding mint’s gene pool that reached the northern Andes, at most leading to three *M. spicata* clusters with two likely independent origins, deemed further consideration. Therefore, we encourage future evolutionary/phylogenetic studies to expand sampling beyond our study region in order to address this assumption more strongly.

From a more technical point of view, we had wished to be able to capture phased haplotypes. This is because haplotype divergence is fundamental to corroborate divergence among clonal organisms at the genomic level. After all, a null prediction is that haplotypes would accumulate mutations independently. Therefore, we invite future researchers to explicitly consider BAC library-cloning strategies as a pre-sequencing step that would enable accurate phase estimation and mutation tracing.

Last but not least, we would have preferred to carry out a full enrichment analysis as typically implemented in model species. Unfortunately, limitedly annotated resources for *M. spicata* precluded an accurate enrichment computation. Therefore, instead of reporting biased enrichment statistics, we opted for a detailed biological discussion of the major GO findings, which surprisingly did not include prominent flavor and flavonoid pathways. Still, we are looking forward to seeing better annotations for mint’s genomic resources in the years to come. This may assist with novel uses of mint’s clonal diversity.

## 4. Materials and Methods

### 4.1. Plant Material

A total of 38 specimens (Table S1) were sampled under protected conditions and free exposure from 14 mint export farms between 2019 and 2020 in the northwest Andes of Colombia, a major region for mint production. Only farms with clonally propagated mint were considered as a way to narrow down founder lineages. Mint production in these farms is mainly intended for exportation to the US fresh market.

According to previously standardized botanical traits [16], 36 of the 38 collected specimens were *M. spicata*, while the remaining two were *M. × piperita*. The identification of these specimens was carried out by farmers and specialized staff of the greenhouse at Universidad Católica de Oriente (UCO). Plant tissues (i.e., stems and roots) were sampled in situ and sent to UCO’s greenhouse for clonal propagation and 9 months of acclimation before sampling leaves for RNA sequencing. Two additional specimens from UCO’s collection were also included as species controls for *M. spicata* and *M. × piperita*.

### 4.2. RNA Extraction and Whole Transcriptome Sequencing

With the intention to recover the highest quantity and quality of mint’s RNA, we carried out a comparative experiment of plant extraction protocols. A total of six different RNA extraction protocols and library preparation methods were tested at the Molecular Genetic Laboratory of AGROSAVIA in Tibaitatá’s Research Station (Colombia) using two random samples of *M. × piperita* and *M. spicata*, in addition to UCO’s reference controls for each species. The six protocols were: (1) In-house AGROSAVIA, (2) In-house AGROSAVIA modified in volumes and time, (3) In-house AGROSAVIA with variations for species with high content of polysaccharides and polyphenols, (4) In-house AGROSAVIA with Trizol, (5) Qiagen^®^ RNeasy Plant Mini Kit (Hilden, Germany) and (6) Qiagen^®^ RNeasy Plant Mini Commercial Kit with Trizol (Hilden, Germany). Of the six tested protocols, the Qiagen^®^ RNeasy Plant Mini Kit commercial kit allowed to obtain a better quality and absorbance ratios of A260/A280 and A260/230.

Quantification of extracted cDNA was done by a spectrophotometry method using the Nanodrop^®^ 2000 equipment (Thermo Fisher Scientific, United States) and by a fluorometric method using the Qubit^®^ dsDNA HS fluorometer (Life Technologies, Sweden). Library construction was performed using the SureSelect Strand-Specific RNA^®^ kit for multiplexed sequencing by Illumina^®^. Then, the libraries were quantified by the fluorometric method using the Qubit^®^ dsDNA HS fluorometer. The concentration and fragment sizes of the cDNA libraries were evaluated using the TapeStation 4200^®^ kit (Agilent Technologies, United States) and the High Sensitivity D1000 kit. DNA sequences were obtained using single-end Illumina 2500 Hiseq (Macrogen, South Korea).

### 4.3. Bioinformatics Processing

In order to clean the sequence data, we carried out a script to automate the software Trimmomatic (Bolger et al. 2014) with the main parameters: ILLUMINACLIP:TruSeq3-SE:2:30:10, SLIDINGWINDOW:4:20 and MINLEN:After that. We performed an analysis of quality from the trimmed *fastq* files in the software FastQC [17] using Illumina 1.9 encoding.

Comparative works [10] have suggested that the most efficient pipeline to reconstruct the matrix of allelic variants from RNA sequencing is the integration of the TRINITY algorithm [11] as a de novo assembler with a GATK [12] protocol for SNP calling. This way, we obtained a de novo transcriptome from the samples of mint by means of the software TRINITY [11] using the platform *Galaxy* v.2.9.1 [18]. A supertranscript was then built as a reference to map and identify allelic polymorphisms in the context of de novo assembly (i.e., without reference genome). The supertranscript was constructed by collapsing regions of unique and common sequences between splice isoforms into a single linear sequence [13]. We finally obtained a supertranscript using TRINITY to be used by GATK4 as a reference in the SNP calling protocol.

From all collected samples, total RNA was successfully extracted and bioinformatically processed for 29 specimens (Table 3) from leaves stored at −80°C using the standardized Qiagen^®^ RNeasy Plant Mini Kit. Our ultimate aim was to identify allelic polymorphisms from these 29 samples of mint, targeting coding regions in an otherwise complex genome structure with presumably abundant polyploidization and hybridization signatures [8].

Therefore, we made a script to automate the process of SNP calling by the protocol GATK4 using the *HaplotypeCaller* function and the algorithm BWA-MEM [19,20]. The mapping statistics were computed using the *flagstat* function from the software *Samtools* v.1.9 [21] using the platform *Galaxy* v.2.9.1 [18]. Rather than relying on suboptimal phase imputation strategies poorly calibrated for mint RNA resources, we opted to carry out the analysis on more conservative allelic variants. The resultant SNP matrix was then filtered with a minimum depth of 20X and a stringent threshold of missing data of 4% using the software TASSEL v.5.2.78 [22]. All scripts, raw data and input files are available in https://github.com/FelipeLopez2019/RNAseq-SNP-Calling-GATK4-Mint.

### 4.4. Genetic Clustering and Putative Somaclonal Variation in Mint

From the identification of allelic polymorphisms consolidated in the SNP matrix, we carried out a prospective analysis of linear dimensional reduction by molecular principal components (PCA) using the *glPca* function of the R *adegenet* v.2.1.4 package [23]. After that, in order to assess the genetic substructure in the *M. spicata* and *M. × piperita* assemblages, we carried out a clustering analysis following a Data Science perspective [24]. Specifically, we ran a script of clustering validation using the function *OptCluster* from the R-package *OptCluster* [25], an optimized version of the function *clValid* [26]. The *OptCluster* function considered these methods: the partitional algorithms *K*-means [27,28] and *k*-medoids [29], and the hierarchical algorithms AGNES (AGglomerative NESting) and DIANA (Divisive analysis, [29]) with Genetic Algorithms and Cross-Entropy validation. We also ran another algorithm for clustering validation called *NbClust* [30]. Finally, we constructed a UPGMA dendrogram with 10,000 bootstrap replicates using the function *aboot* of the R-package *poppr* [31] and Nei’s distance [32].

### 4.5. Gene Functionality of Polymorphic Transcripts

With the goal of obtaining the ontological analysis of polymorphic coding variation in mint, all polymorphic transcripts were analyzed by means of the software *Blast2GO* [33] using the algorithm *BlastX* with the *nr* database, threshold E-value of 1.0 × 10^−6^, word size of 6, and 10 *hits* per transcript. Furthermore, we carried out the GO mapping from the hits of BLASTX to obtain functional labels using the GO Gene Annotation files and the *Uniprot* database. The annotation process was generated to select GO terms from the GO pool obtained in the previous step and assign them to the query sequence. We ran the annotation process using the taxonomy filter of ‘flowering plants’ [Magnoliopsida], a value of 55 as annotation cut-off, 5 as GO weight and a mean of convergence of 80% and 100% for computational analysis and experimental evidence, respectively. In order to sort the GO outputs in hierarchical pathways, we generated tree diagrams of biological, cellular and molecular processes using 150 as *Nodescore* filter, indicating the high associativity of GO ID with the mint transcripts. Finally, we carried out the KEEG pathway analysis using the *GO-EnzymeCode* function in *Blast2GO* (https://www.genome.jp/kegg/pathway.html, accessed on 30 March 2022) with the aim of exploring the metabolic pathways more prone to nucleotide diversity in mint.

## 5. Conclusions

Coding polymorphism in *M. spicata* from northwest South America likely traced back to two independent origins that were clonally propagated in the region ever since. Putative somaclonal variants were mostly found in primary metabolic pathways, indicating a plausible predominant role of silent mutations. However, co-opted adaptive variants may still be expected as part of mint’s introduction and adaptation to the northern Andes, where it had to face shorter day length and unexpected herbivore pressures. These hypotheses on putative somatic mutations deem further study. Methodologically, the pipeline RNA-Seq + TRINITY + GATK4 + *Blast2GO* helps characterize somaclonal variation and associated metabolic pathways in complex genomes.

## Figures and Tables

**Table 1:** Mapping statistics against the supertranscript. Mapping of each specimen’s transcript profile used as reference the supertranscript and the GATK4 protocol. The total number of sequences per sample, mapped sequences, duplicates and purified sequences in the refinement of the protocol are shown in this table. For details on the sampling information of each specimen please refer to Materials and Methods.

Sample ID	Total Transcripts	Supplementary Transcripts	Duplicate Transcripts	Mapped Transcripts	Unmapped Duplicates	Mapped Ratio
MLV20-15244	13,595,799	438,550	10,261,916	13,431,369	3,169,453	0.988
MLV20-15245	13,893,704	422,388	9,887,386	13,560,195	3,672,809	0.976
MLV20-15246	19,286,261	628,197	14,805,807	18,976,542	4,170,735	0.984
MLV20-15247	13,264,697	388,203	9,755,435	12,984,582	3,229,147	0.979
MLV20-15248	14,873,365	403,064	10,434,595	14,174,092	3,739,497	0.953
MLV20-15249	11,814,041	375,642	8,206,501	11,568,380	3,361,879	0.979
MLV20-15250	12,501,455	260,480	9,220,160	11,900,514	2,680,354	0.952
MLV20-15251	12,138,901	348,593	8,715,938	11,859,700	3,143,762	0.977
MLV20-15252	15,738,616	474,121	11,982,280	15,212,909	3,230,629	0.967
MLV20-15253	13,479,991	429,102	9,136,387	13,252,005	4,115,618	0.983
MLV20-15254	14,800,126	468,816	10,804,162	14,579,876	3,775,714	0.985
MLV20-15255	15,818,146	444,333	11,731,011	15,593,508	3,862,497	0.986
MLV20-15256	15,727,298	509,431	11,699,111	15,532,158	3,833,047	0.988
MLV20-15257	21,828,779	704,838	17,154,954	21,564,209	4,409,255	0.988
MLV20-15258	14,676,563	452,082	10,462,672	14,444,502	3,981,830	0.984
MLV20-15259	14,468,839	474,835	10,691,456	14,284,865	3,593,409	0.987
MLV20-15260	15,790,191	453,092	9,426,258	12,650,256	3,223,998	0.801
MLV20-15261	9,381,824	276,062	5,290,265	8,045,530	2,755,265	0.858
MLV20-15262	15,512,116	493,130	10,567,253	15,326,192	4,758,939	0.988
MLV20-15263	20,188,093	592,807	14,099,354	19,789,887	5,690,533	0.98
MLV20-15264	12,867,595	441,468	8,952,424	12,227,682	3,275,258	0.95
MLV20-15265	7,777,046	231,316	4,725,157	7,386,866	2,661,709	0.95
MLV20-15266	20,521,417	686,319	15,064,734	20,332,633	5,267,899	0.991
MLV20-15267	20,002,666	669,043	14,947,013	19,630,558	4,683,545	0.981
MLV20-15268	24,788,824	718,176	19,701,023	24,412,087	4,711,064	0.985
MLV20-15269	19,153,299	556,314	14,032,872	18,839,624	4,806,752	0.984
MLV20-15270	11,730,613	372,805	8,680,285	11,611,304	2,931,019	0.99
MLV20-15271	16,841,331	556,417	11,787,644	16,511,475	4,723,831	0.98
MLV20-15272	19,610,849	640,811	14,233,830	19,274,477	5,040,647	0.983

**Table 2:** Related KEEG pathways (52) across transcripts using enzyme codes in *Blas2GO* outputs.

KEGG Pathway	# of Transcripts	# of Enzymes	KEEG Label
Glycolysis/Gluconeogenesis	15	16	Carbohydrate metabolism
Cysteine and methionine metabolism	13	13	Amino acid metabolism
Carbon fixation in photosynthetic organisms	11	13	Energy metabolism
Methane metabolism	10	10	Energy metabolism
Pyruvate metabolism	9	12	Carbohydrate metabolism
Glycine, serine and threonine metabolism	9	9	Amino acid metabolism
Glyoxylate and dicarboxylate metabolism	9	9	Carbohydrate metabolism
Starch and sucrose metabolism	8	8	Carbohydrate metabolism
Galactose metabolism	7	8	Carbohydrate metabolism
Nitrogen metabolism	7	8	Energy metabolism
Citrate cycle (TCA cycle)	7	7	Carbohydrate metabolism
Oxidative phosphorylation	7	7	Energy metabolism
Tyrosine metabolism	7	7	Amino acid metabolism
Amino sugar and nucleotide sugar metabolism	6	7	Carbohydrate metabolism
Phenylalanine, tyrosine and tryptophan biosynthesis	6	7	Amino acid metabolism
Terpenoid backbone biosynthesis	6	7	Metabolism of terpenoids and polyketides
Carbon fixation pathways in prokaryotes	6	6	Energy metabolism
Glycerolipid metabolism	6	6	Lipid metabolism
Pentose phosphate pathway	6	6	Carbohydrate metabolism
Alanine, aspartate and glutamate metabolism	5	7	Amino acid metabolism
Tryptophan metabolism	5	5	Amino acid metabolism
Ubiquinone and other terpenoid-quinone synthesis	5	5	Metabolism of cofactors and vitamins
Ascorbate and aldarate metabolism	5	4	Carbohydrate metabolism
Glutathione metabolism	5	4	Metabolism of other amino acids
Phenylalanine metabolism	4	5	Amino acid metabolism
Phenylpropanoid biosynthesis	4	5	Biosynthesis of other secondary metabolites
alpha-Linolenic acid metabolism	4	4	Lipid metabolism
Fructose and mannose metabolism	4	4	Carbohydrate metabolism
O-Antigen nucleotide sugar biosynthesis	4	4	Glycan biosynthesis and metabolism
Porphyrin metabolism	4	4	Metabolism of cofactors and vitamins
Cyanoamino acid metabolism	3	4	Metabolism of other amino acids
Inositol phosphate metabolism	3	4	Carbohydrate metabolism
Pentose and glucuronate interconversions	3	4	Carbohydrate metabolism
Glycerophospholipid metabolism	3	3	Lipid metabolism
Selenocompound metabolism	3	3	Metabolism of other amino acids
Arginine biosynthesis	2	3	Amino acid metabolism
Carotenoid biosynthesis	2	2	Metabolism of terpenoids and polyketides
Drug metabolism—cytochrome P450	2	2	Xenobiotics biodegradation and metabolism
Drug metabolism—other enzymes	2	2	Xenobiotics biodegradation and metabolism
Fatty acid degradation	2	2	Lipid metabolism
Isoquinoline alkaloid biosynthesis	2	2	Biosynthesis of other secondary metabolites
Lysine degradation	2	2	Amino acid metabolism
Metabolism of xenobiotics by cytochrome P450	2	2	Xenobiotics biodegradation and metabolism
Nicotinate and nicotinamide metabolism	2	2	Metabolism of cofactors and vitamins
One carbon pool by folate	2	2	Metabolism of cofactors and vitamins
Propanoate metabolism	2	2	Carbohydrate metabolism
Steroid biosynthesis	2	2	Metabolism of terpenoids and polyketides
Styrene degradation	2	2	Xenobiotics biodegradation and metabolism
Sulfur metabolism	2	2	Energy metabolism
Thiamine metabolism	2	2	Metabolism of cofactors and vitamins
Tropane, piperidine and pyridine alkaloid synthesis	2	2	Biosynthesis of other secondary metabolites
Valine, leucine and isoleucine degradation	2	2	Amino acid metabolism

**Table 3:** *Mentha* spp. used for RNA-Seq and sampling localities in Colombia, Antioquia province.

Sequencing ID	ID UBV UCO	Species	Conditions	Municipality	Latitude	Longitude	Elevation
MLV20-15244_s	1	*M. spicata*	Open field	La Ceja	6.067500	−75.415056	2208
MLV20-15245_s	2	*M. spicata*	Open field	La Ceja	6.067500	−75.415056	2208
MLV20-15246_s	3	*M. spicata*	Protected cultivation	El Retiro	6.066111	−75.449528	2218
MLV20-15247_s	4	*M. spicata*	Open field	El Retiro	6.066111	−75.449528	2218
MLV20-15248_s	5	*M. spicata*	Protected cultivation	Medellin	6.198222	−75.511889	2554
MLV20-15249_s	6	*M. spicata*	Protected cultivation	Medellin	6.198222	−75.511889	2554
MLV20-15250_s	10	*M. spicata*	Open field	Marinilla	6.167222	−75.325861	2131
MLV20-15251_s	11	*M. spicata*	Open field	Marinilla	6.167222	−75.325861	2131
MLV20-15252_s	12	*M. piperita*	Protected cultivation	Rionegro	6.192972	−75.360972	2128
MLV20-15253_s	14	*M. spicata*	Open field	Rionegro	6.192972	−75.360972	2128
MLV20-15254_s	15	*M. spicata*	Open field	Rionegro	6.198111	−75.350639	2118
MLV20-15255_s	16	*M. spicata*	Open field	Rionegro	6.198111	−75.350639	2118
MLV20-15256_s	17	*M. spicata*	Protected cultivation	El Retiro	6.066111	−75.449528	2218
MLV20-15257_s	18	*M. spicata*	Protected cultivation	Medellin	6.198222	−75.511889	2554
MLV20-15258_s	19	*M. spicata*	Protected cultivation	Medellin	6.198222	−75.511889	2554
MLV20-15259_s	20	*M. spicata*	Protected cultivation	Medellin	6.198222	−75.511889	2554
MLV20-15260_s	21	*M. spicata*	Open field	Rionegro	6.067500	−75.415056	2208
MLV20-15261_s	22	*M. spicata*	Open field	Rionegro	6.067500	−75.415056	2208
MLV20-15262_s	24	*M. spicata*	Protected cultivation	La Unión	5.910333	−75.412278	2277
MLV20-15263_s	25	*M. spicata*	Protected cultivation	La Unión	5.910333	−75.412278	2277
MLV20-15264_s	26	*M. spicata*	Protected cultivation	San Vicente Ferrer	6.325194	−75.335694	2251
MLV20-15265_s	27	*M. spicata*	Protected cultivation	San Vicente Ferrer	6.325194	−75.335694	2251
MLV20-15266_s	28	*M. spicata*	Protected cultivation	San Vicente Ferrer	6.325194	−75.335694	2251
MLV20-15267_s	29	*M. piperita*	Protected cultivation	Carmen de Viboral	6.082287	−75.316147	2260
MLV20-15268_s	33	*M. spicata*	Protected cultivation	Carmen de Viboral	6.082287	−75.316147	2260
MLV20-15269_s	44	*M. spicata*	Protected cultivation	Guarne	6.213960	−75.417330	2200
MLV20-15270_s	45	*M. spicata*	Protected cultivation	Medellin	6.198222	−75.511889	2554
MLV20-15271_s	Reference piperita	*M. piperita*	Protected cultivation	-	-	-	2118
MLV20-15272_s	Reference spicata	*M. spicata*	Protected cultivation	-	-	-	2118

## Data Availability

Raw data, input files and bioinformatic scripts are available in https://github.com/FelipeLopez2019/RNAseq-SNP-Calling-GATK4-Mint. Data supporting reported results can be found is the Supplementary Materials. Any further details can be obtained per direct request to the corresponding authors.

## Supplementary Materials

The following supporting information can be downloaded at: https://www.mdpi.com/article/10.3390/ijms23105291/s1, **Table S1**. A total of 38 specimens were sampled under protected conditions and free exposure from 14 mint export farms during 2019 and 2020 in the northwest Andes (Antioquia province, Colombia). **Table S2**. RNA extraction was carried out across 29 specimens, retrieving an optimal state of RNA conservation and health, with a mean RNA concentration of 922.02 ug/uL (IC: 236.51) using Nanodrop^®^, a mean RNA concentration of 94.79 ug/uL (CI: 8.78) using Qubit^®^, mean A260/280 ratio of 2.110 (CI: 0.016), and mean A260/230 ratio of 2.015 (CI: 0.229). **Table S3**. Across all specimens, electropherogram suggested an ideal distribution of fragments with defined peaks, and the absence of contaminants. **Table S4**. *Blas2GO* outputs using all 912 transcripts derived from the supertranscript. Among these, 96.10% obtained hits in *BlastX* using the software *Blast2GO*. The 84.57% of these Blast hits were mapped, and the 94.27% of the mapped results were annotated with GO and Enzyme codes. **Table S5**. KEEG pathways associated to all transcripts from enzymes codes of *Blas2GO* outputs. **Figure S1**. *MultiFastQC* analysis for all specimens, with: (a) a quality score greater than 30 using 1.9 Illumina encoding, (b) a moderate duplications percentage, (c) a deviation of GC percentage to be expected in RNAseq data, and (d) absence of adapters. **Figure S2**. Supertranscript assembly. (a) De novo assembly of transcriptome using the software TRINITY from all trimmed *fastq*, composed of 509,754 transcripts with an average length of 557.9 bp, a minimum of 178 bp, and a maximum of 12,186 bp. (b) The supertranscript of mint presented 352,512 transcripts with an average length 472.2 pb, a minimum of 201 bp, and a maximum of 15,765 bp. **Figure S3**. Multidimensional Venn diagram depicting the overlap among transcript variants. **Figure S4**. GO pathway to analyze polymorphic cellular processes using GO codes from Blast2GO. **Figure S5**. GO pathway to analyze polymorphic molecular processes via GO codes from Blast2GO. **Figure S6**. Main KEGG pathway of carbohydrate metabolism for glycoly-sis/Gluconeogenesis synthesis using the enzymes codes from *Blast2GO* outputs. **Figure S7**. Main KEGG pathway of amino acid metabolism associated to cysteine and methionine synthesis.
