# Supplementary material for: Whole Transcriptome Sequencing Unveils the Genomic Determinants of Putative Somaclonal Variation in Mint (Mentha L.)
Source: Int J Mol Sci. 2022 May 10;23(10):5291. doi: 10.3390/ijms23105291 (PMC9141282; doi:10.3390/ijms23105291)
Supplement: Supplementary file 1 [file ijms-23-05291-s001.zip › Submitted_Supp_v3/Figure S1.pdf]

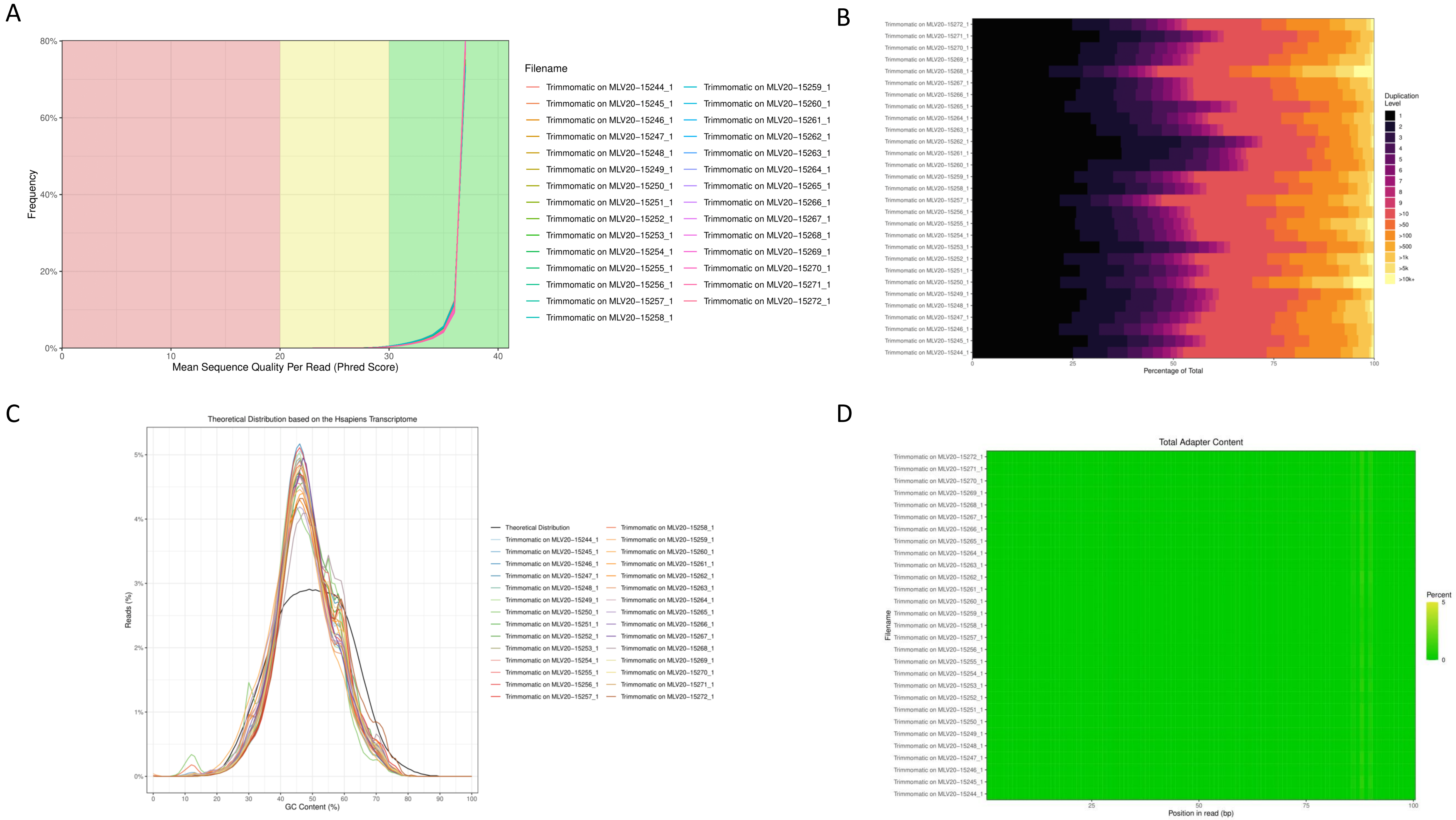

A
80%
60%
40%
20%
0%
Frequency
0
10
20
30
40
Mean Sequence Quality Per Read (Phred Score)
Filename
Trimmomatic on MLV20−15244_1
Trimmomatic on MLV20−15245_1
Trimmomatic on MLV20−15246_1
Trimmomatic on MLV20−15247_1
Trimmomatic on MLV20−15248_1
Trimmomatic on MLV20−15249_1
Trimmomatic on MLV20−15250_1
Trimmomatic on MLV20−15251_1
Trimmomatic on MLV20−15252_1
Trimmomatic on MLV20−15253_1
Trimmomatic on MLV20−15254_1
Trimmomatic on MLV20−15255_1
Trimmomatic on MLV20−15256_1
Trimmomatic on MLV20−15257_1
Trimmomatic on MLV20−15258_1
Trimmomatic on MLV20−15259_1
Trimmomatic on MLV20−15260_1
Trimmomatic on MLV20−15261_1
Trimmomatic on MLV20−15262_1
Trimmomatic on MLV20−15263_1
Trimmomatic on MLV20−15264_1
Trimmomatic on MLV20−15265_1
Trimmomatic on MLV20−15266_1
Trimmomatic on MLV20−15267_1
Trimmomatic on MLV20−15268_1
Trimmomatic on MLV20−15269_1
Trimmomatic on MLV20−15270_1
Trimmomatic on MLV20−15271_1
Trimmomatic on MLV20−15272_1
B
Percentage of Total
0
25
50
75
100
Duplication Level
1
2
3
4
5
6
7
8
9
>10
>50
>100
>500
>1k
>5k
>10k+
C
Theoretical Distribution based on the Hsapiens Transcriptome
Reads (%)
GC Content (%)
Theoretical Distribution
D
Total Adapter Content
Filename
Position in read (bp)
25
50
75
100
Percent
5
0
