# Supplementary figures and images for: Whole Transcriptome Sequencing Unveils the Genomic Determinants of Putative Somaclonal Variation in Mint (Mentha L.)
Source: Int J Mol Sci. 2022 May 10;23(10):5291. doi: 10.3390/ijms23105291 (PMC9141282; doi:10.3390/ijms23105291)

**A**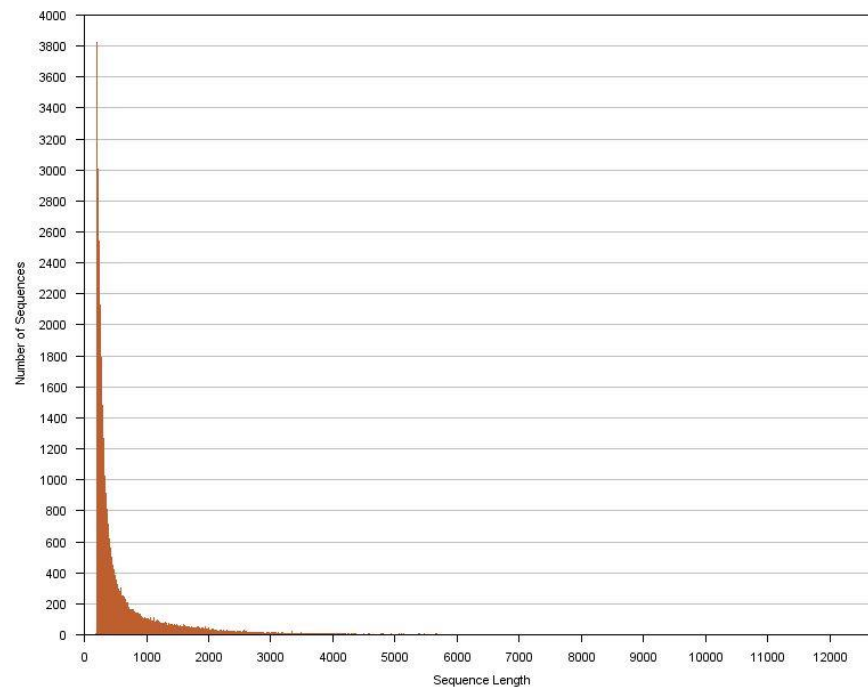**B**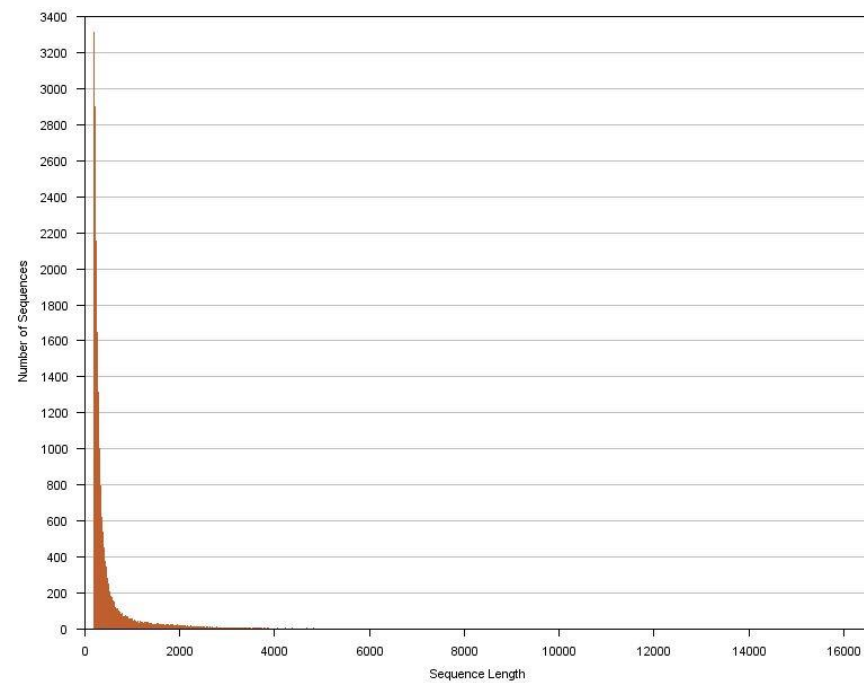

Supplement: Supplementary file 1 [file ijms-23-05291-s001.zip › Submitted_Supp_v3/Figure S2.pdf]

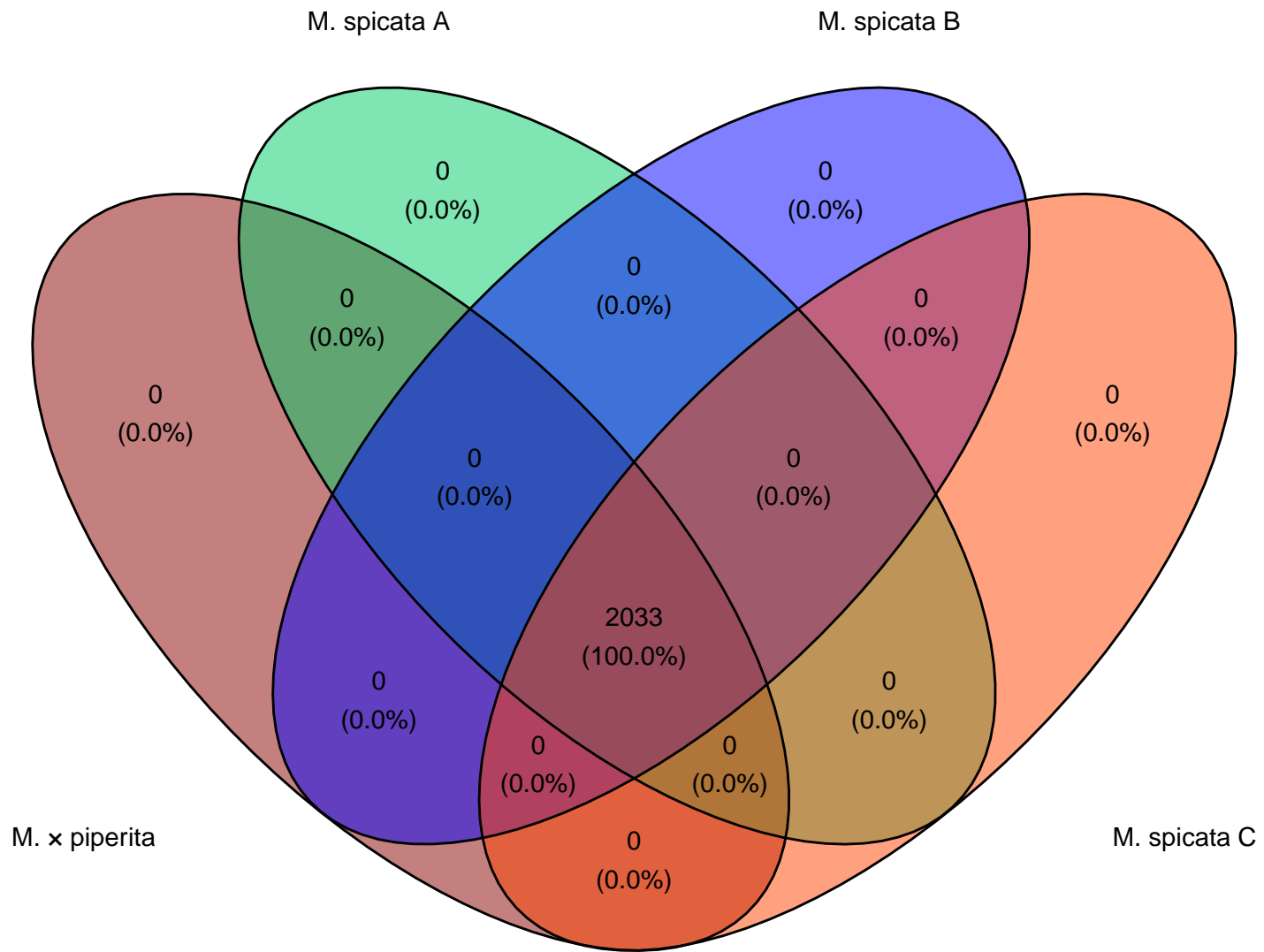

Supplement: Supplementary file 1 [file ijms-23-05291-s001.zip › Submitted_Supp_v3/Figure S3.pdf]

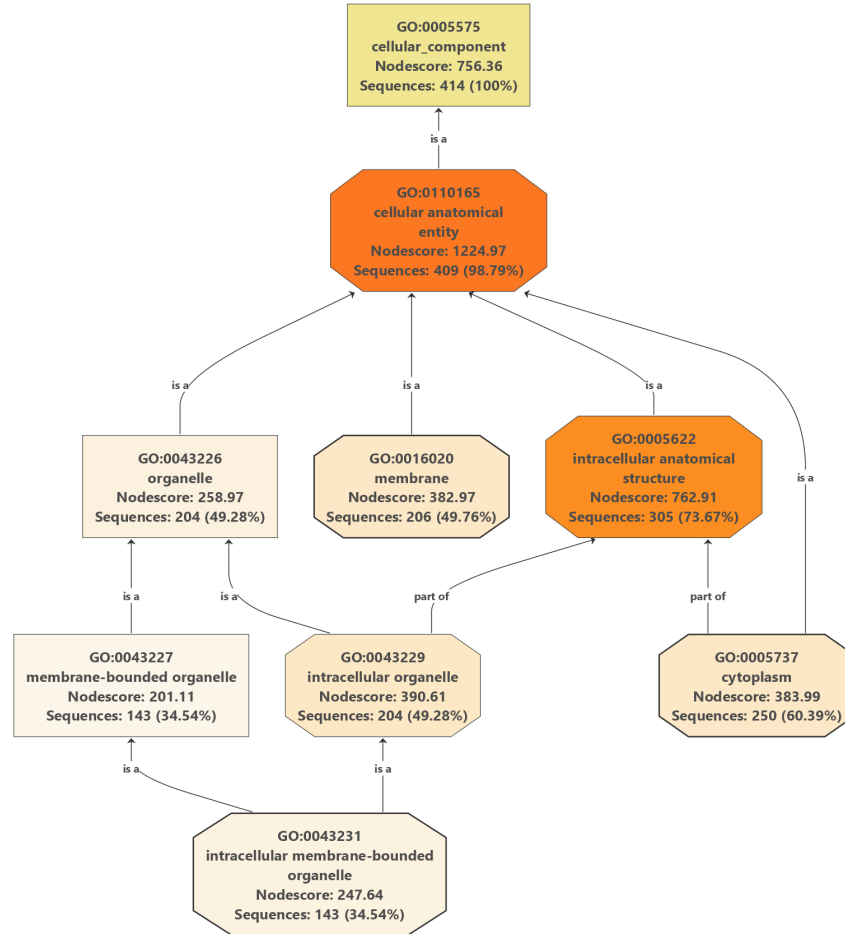

Supplement: Supplementary file 1 [file ijms-23-05291-s001.zip › Submitted_Supp_v3/Figure S4.pdf]

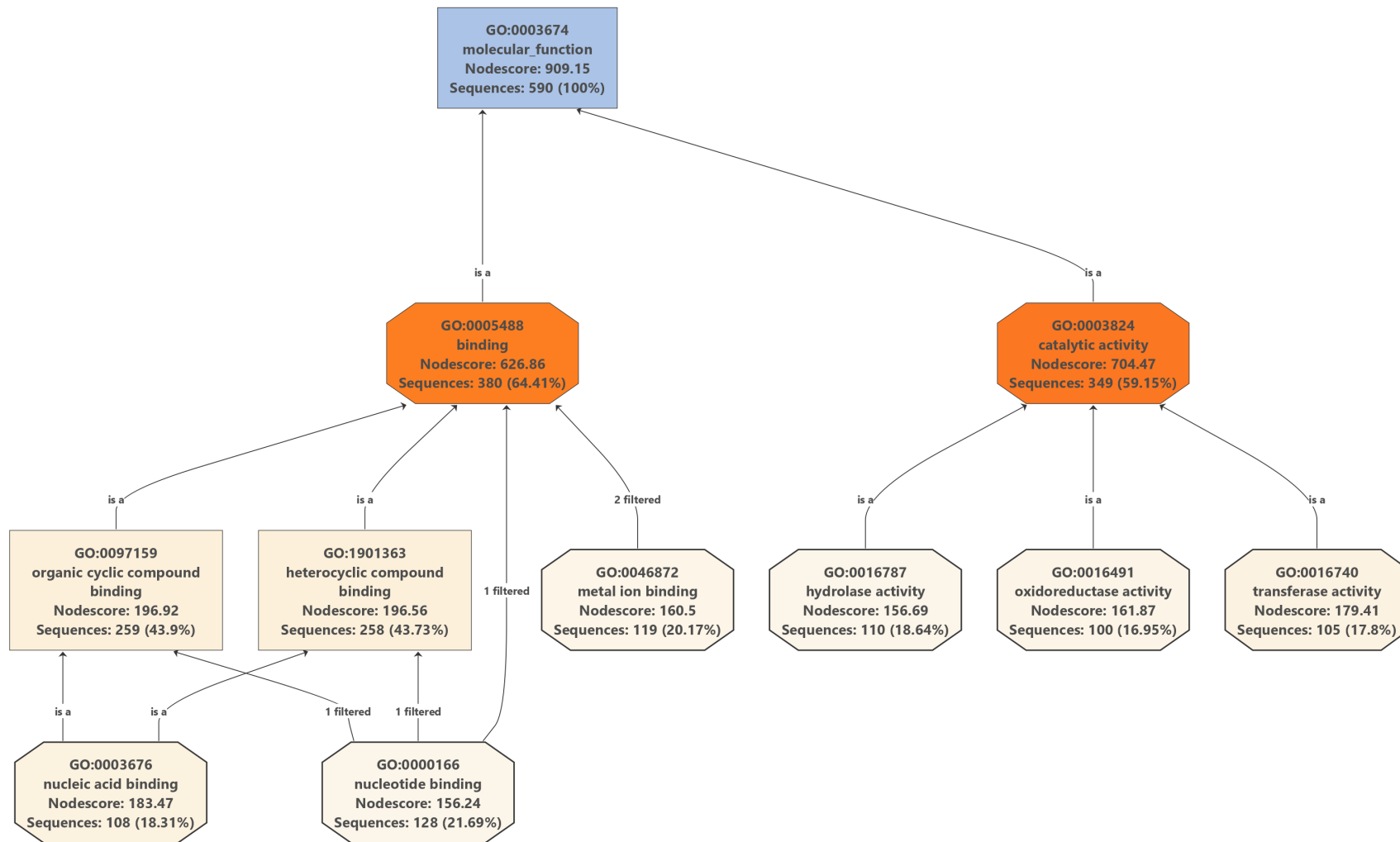

Supplement: Supplementary file 1 [file ijms-23-05291-s001.zip › Submitted_Supp_v3/Figure S5.pdf]

# GLYCOLYSIS / GLUCONEOGENESIS

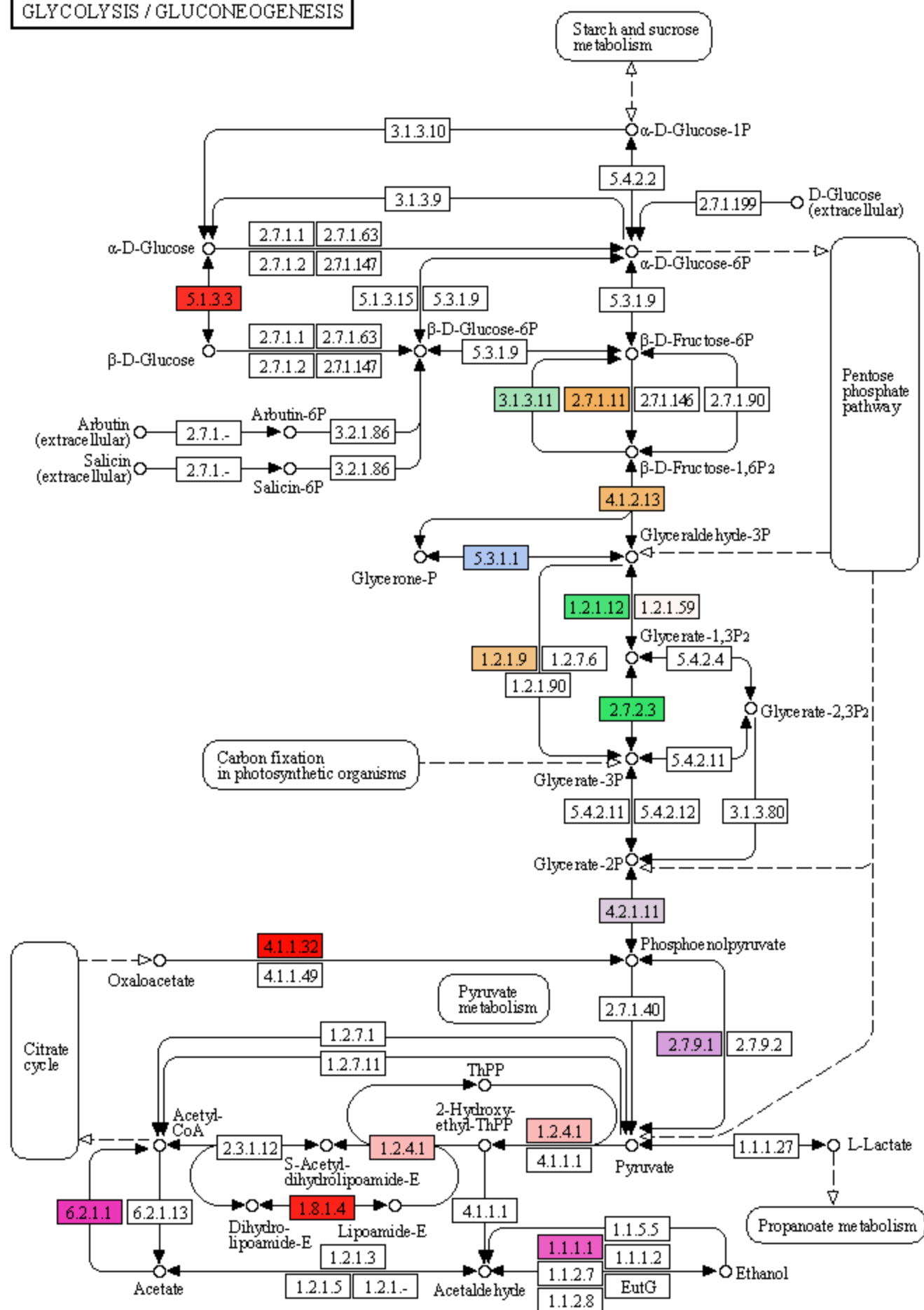

Supplement: Supplementary file 1 [file ijms-23-05291-s001.zip › Submitted_Supp_v3/Figure S6.pdf]
