# Supplementary material for: Whole Transcriptome Sequencing Unveils the Genomic Determinants of Putative Somaclonal Variation in Mint (Mentha L.)
Source: Int J Mol Sci. 2022 May 10;23(10):5291. doi: 10.3390/ijms23105291 (PMC9141282; doi:10.3390/ijms23105291)
Supplement: Supplementary file 1 [file Figure S7.pdf › Submitted_Supp_v3/Figure S7.pdf]

CYSTEINE AND METHIONINE METABOLISM

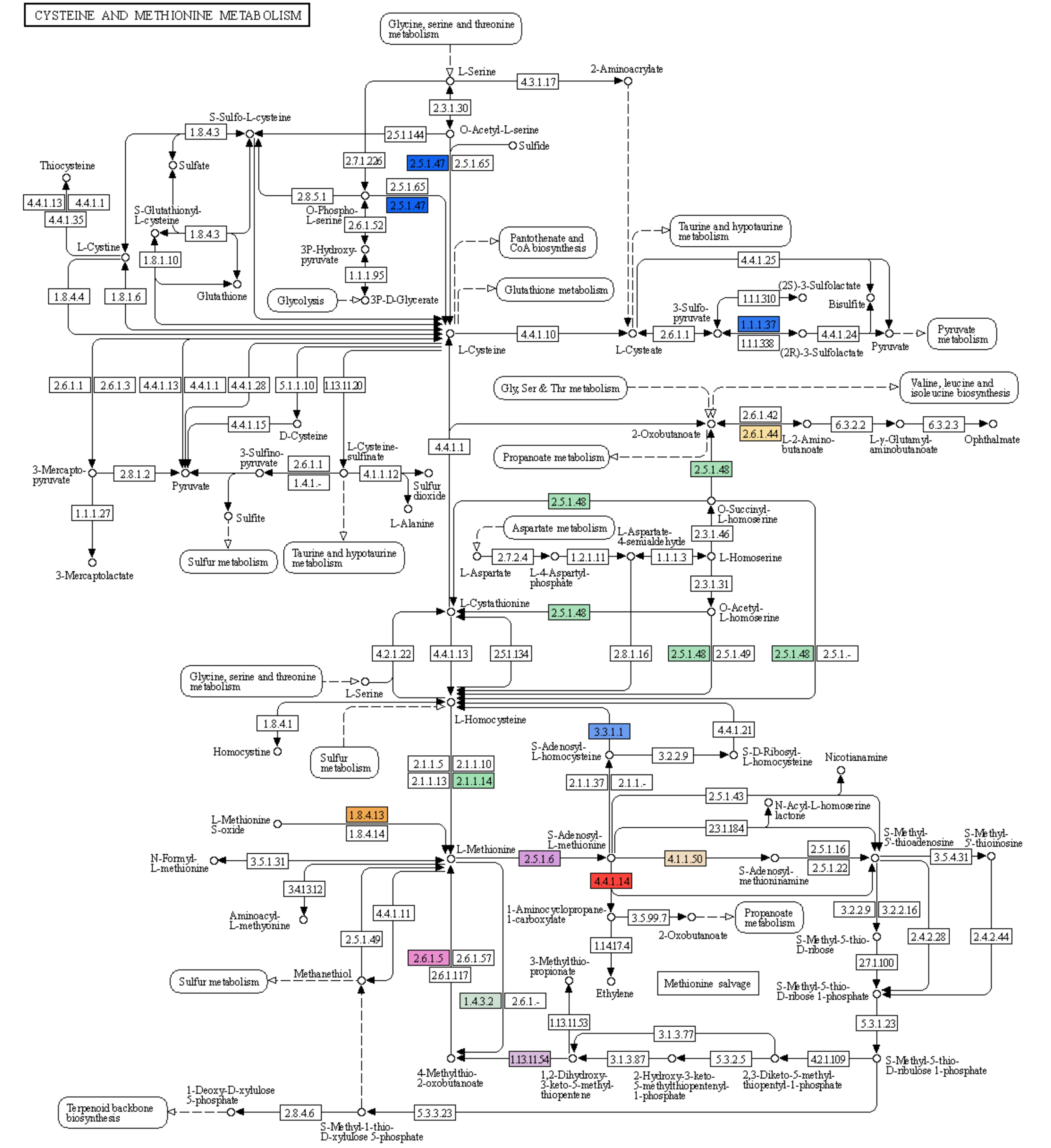

00270 1/19/22
(c) Kanehisa Laboratories
